# Unveiling a sudden unexplained death case by whole exome sequencing and bioinformatic analysis

**DOI:** 10.1002/mgg3.1182

**Published:** 2020-02-26

**Authors:** Martina Modena, Vincenzo Castiglione, Paolo Aretini, Chiara M. Mazzanti, Enrica Chiti, Alberto Giannoni, Michele Emdin, Marco Di Paolo

**Affiliations:** ^1^ Institute of Life Sciences Scuola Superiore Sant'Anna Pisa Italy; ^2^ Fondazione Pisana per la Scienza ONLUS Pisa Italy; ^3^ Cardiology Division University of Pisa Pisa Italy; ^4^ Fondazione Toscana Gabriele Monasterio Pisa Italy; ^5^ Institute of Legal Medicine University of Pisa Pisa Italy

**Keywords:** arrhythmia, bioinformatics, sudden cardiac death, whole exome sequencing

## Abstract

**Background:**

Sudden unexplained death (SUD) refers to cases of sudden death where autopsy fails to identify any cardiac or extracardiac underlying cause. Guideline‐directed standard genetic testing identifies a disease‐causing mutation in less than one‐third of cases of SUD. Conversely, whole exome sequencing (WES) may provide the key to solve most cases of SUD even after several years from the subject's death.

**Methods:**

We report on a case of sudden unexpected death of a 37‐year‐old male, with inconclusive autopsy conducted 14 years ago. A recent reevaluation through WES was performed on DNA extracted from left ventricular samples. A multiple step process including several “in silico” tools was applied to identify potentially pathogenic variants. Data analysis was based on a 562 gene panel, including 234 candidate genes associated with sudden cardiac death or heart diseases, with the addition of 328 genes highly expressed in the heart. WebGestalt algorithms were used for association enrichment analysis of all genes with detected putative pathogenic variants.

**Results:**

WES analysis identified four potentially pathogenic variants: RYR2:c.12168G>T, TTN:c.11821C>T (rs397517804), MYBPC3:c.1255C>T (rs368770848), and ACADVL:c.848T>C (rs113994167). WebGestalt algorithms indicated that their combination holds an unfavorable arrhythmic susceptibility which conceivably caused the occurrence of the events leading to our subject's sudden death.

**Conclusion:**

Associating WES technique with online prediction algorithms may allow the recognition of genetic mutations potentially responsible for otherwise unexplained deaths.

## Graphical abstract

We report on a case of sudden unexpected death of a 37‐year‐old male, with inconclusive autopsy conducted 14 years ago, where recent reevaluation through whole exome sequencing (WES) and WebGestalt algorithms allowed the recognition of four genetic mutations (RYR2‐K4056N, TTN‐R3941C, MYBPC3‐R419C, and ACADVL‐V283A) potentially responsible for the subject's sudden death.

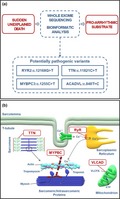

## 1 INTRODUCTION

Sudden death is defined as an unexpected fatal event that occurs within 1 hr of onset of symptoms in an apparently healthy individual or in someone known to be in good health up to 24 hr before the event (Priori et al., 2015). Up to 85% of cases are represented by sudden cardiac death (SCD), which has an estimated incidence of 1 to 10 cases/100,000 people/year (Hayashi, Shimizu, & Albert, 2015). Most cases of SCD in young adults are related to inherited cardiac diseases (Saenen et al., 2015). When sudden death occurs and autopsy fails to identify any cardiac or extracardiac underlying cause, the case is classified as sudden unexplained death (SUD). A genetic testing is recommended to establish the diagnosis, starting from four common channelopathy‐causative genes (KCNQ1, Mendelian Inheritance in Man (MIM) number: 607,542; KCNH2, MIM number: 152,427; SCN5A, MIM number: 600,163; RYR2, MIM number: 180,902), and then extending the assessment to up to 10 genes (Ackerman et al., 2011). This protocol allows to identify a disease‐causing mutation in less than one‐third of cases of SUD, which are then reclassified as SCD. Similar results have been obtained in studies applying next‐generation sequencing (NGS) technique with a panel of genes up to 192 (Bagnall et al., 2016; Neubauer et al., 2018; Tester, Medeiros‐Domingo, Will, Haglund, & Ackerman, 2012). However, whole exome sequencing (WES) filtering for a higher number of genes combined with functional enrichment analysis tool could increase the possibility to identify novel candidate genes or combination of genes potentially responsible for the observed phenotype providing the key to solve a larger number of cases of SUD.

## 2 CASE REPORT

A 37‐year‐old male construction worker was found dead in his bed 1 hr after lunchtime on August 2005. External examination showed no traumatic injury, and toxicological blood screen was negative. His clinical history was unremarkable. The heart weighed 350 g (reference average value (Molina & DiMaio, 2012) in men 331 ± 57 g) and had regular morphology and volume. At microscopic examination, some small foci of interstitial fibrosis were found, but deemed not significant. The other organs did not display any significant pathological abnormality. The dramatic event was then dismissed as a case of SUD.

## 3 MATERIALS AND METHODS

### 3.1 Ethical compliance

The study was performed according to the declaration of Helsinki and was approved by local ethics committee.

### 3.2 Genomic analysis

The case was reevaluated in 2019. DNA was extracted from formalin‐fixed and paraffin‐embedded (FFPE) left ventricular tissue, stored at room temperature, using the Maxwell 16 LEV DNA FFPE Purification Kit (Promega). DNA library preparation and exome capture were performed using the Illumina TruSeq DNA library preparation kit (Illumina) and IDT's xGen exome enrichment kit. DNA libraries were sequenced on an Illumina 500 instrument (Illumina Inc.). Putative pathogenic variants were confirmed by Sanger sequencing.

### 3.3 Bioinformatic analyses

ENLIS Genome Research software was used to annotate and filtrate variants on variant call format (VCF) files generated after primary analysis performed using the Seqmule pipeline, “SeqMule: Automated pipeline for analysis of human exome/genome sequencing data (Guo, Ding, Shen, Lyon, & Wang, 2015).” Variants with a quality score > 30 and a read depth > 20 at the changed position affecting the protein structure were considered. The remaining high‐quality filtered reads were aligned to the human reference sequence (GRch37). The pathogenicity of missense mutations was derived from the DANN (Deleterious Annotation of genetic variants using Neural Networks) model (Quang, Chen, & Xie, 2015) included in the ENLIS Genome Research software. A further “n silico” analysis was performed using MutationTaster (http://www.mutationtaster.org/), FATHMM (http://fathmm.biocompute.org.), MutationAssessor (http://mutationassessor.org), PolyPhen‐2 (http://genetics.bwh.harvard.edu/pph2/), SIFT (http://sift.jcvi.org/), and PROVEAN (http://provean.jcvi.org/index.php). Rare variants were defined as those with a frequency < 0.1% in the general population as reported in gnomAD database (http://gnomad.broadinstitute.org). The American College of Medical Genetics (ACMG) 2015 criteria (Richards, Aziz, Bale, Bick, & Das, 2015) were used to classify identified variants as pathogenic, likely pathogenic, or variant of uncertain significance (VUS). Variants were also verified on ClinVar (https://www.ncbi.nlm.nih.gov/clinvar/), OMIM (https://www.omim.org/), and VarSome (https://varsome.com/) databases. WEB‐based GEne SeT AnaLysis Toolkit (http://www.webgestalt.org/option.php) was used for association enrichment analysis of all genes with detected putative pathogenic variants; p‐values with false discovery rate (FDR) <0.05 were deemed significant. Overrepresentation Enrichment Analysis (ORA) was used as method for enrichment analysis on WebGestalt. Human Phenotype Ontology was selected as functional database (enrichment categories: Phenotype_Human_Phenotype_Ontology). The following parameters were applied for the enrichment analysis: Minimum number of Entrez Gene IDs in the category: Five; maximum number of Entrez Gene IDs in the category: 2000; false discovery rate (FDR) method: Benjamini–Hochberg; significance level: Top 10.

## 4 RESULTS

A multiple step process was applied to identify potentially pathogenic variants. Within the total WES data 13,520 variants were obtained. To ensure good reliability of the variant call, we have only considered mutations found in genetic regions with coverage greater than 20X, although we have searched for the presence of mutations also in low coverage regions, if these were in target genes. Data analysis was based on a 562 gene panel, including 234 candidate genes associated with sudden cardiac death or heart diseases, with the addition of 328 genes highly expressed in the heart (data from proteinatlas.org). After exclusion of common (minor allele frequency (MAF) ≥0.1%) and synonymous variants that did not change the amino acid sequence, 18 heterozygous missense mutations remained (Table S1). We then prioritized the remaining variants based on the ACMG and ClinVar classifications, in silico tools to determine pathogenicity (DANN, Mutation Taster, FATHMM, Mutation Assessor, PolyPhen‐2, SIFT, and PROVEAN) and conservation of the affected amino acid. Eleven variants were predicted as pathogenic by at least four prediction tools (Table S2): a) RYR2:c.12168G>T and RBFOX1: c.1079C>T, which had not been previously described; b) Six variants which had been previously described in the ClinVar Database (TTN:c.11821C>T, rs397517804; FLNC:c.4991C>T, rs780829334; MYBPC3:c.1255C>T, rs368770848; ACADVL:c.848T>C, rs113994167; IL25:c.424C>T, rs1124053; GCOM1:c.91C>A, rs142253131); c) Three single nucleotide variations without a clear clinical significance SCN7A:c.4865G>A (rs188781935), MYO6:c.1852C>T (rs755596824), and APOB:c.2258G>A (rs148502464).

**Figure 1:**
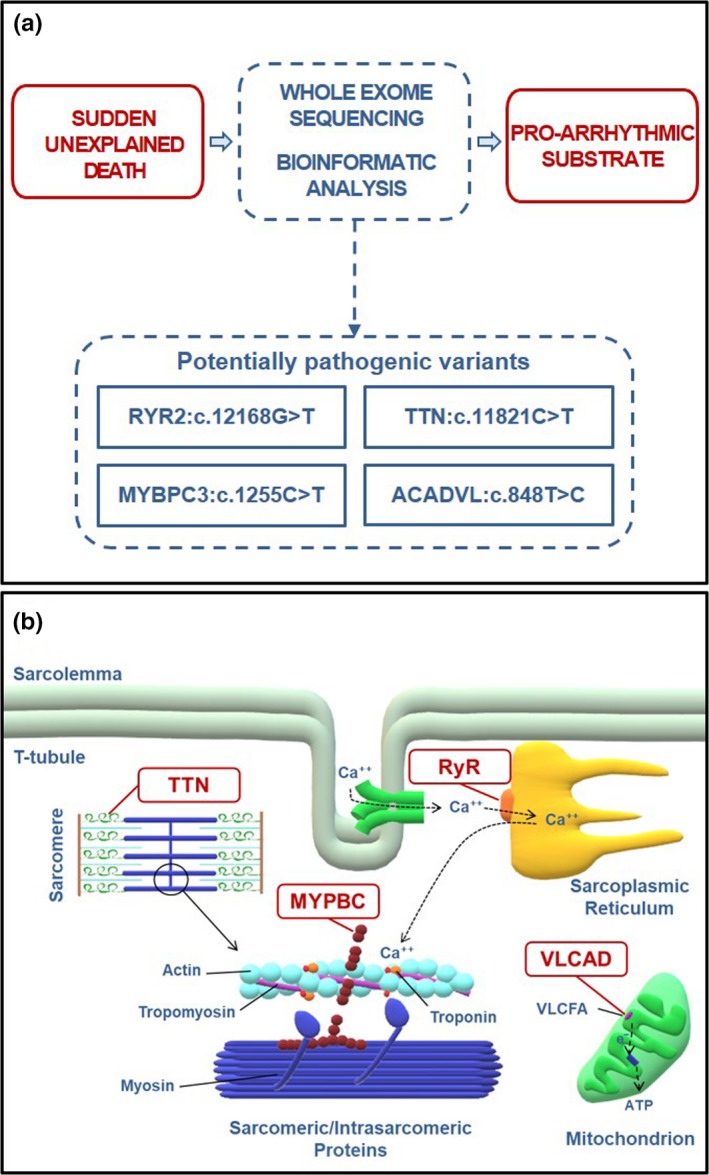
Whole exome sequencing and bioinformatic analyses for identification of the probable cause of death in a case of sudden unexplained death. (a) According to WebGestalt algorithms, the combination of mutations in MYBPC3, ACADVL, TTN, and RYR2 may have created the pro‐arrhythmic substrate responsible for the occurrence of the events leading to our subject's sudden unexplained death. (b) Mutated proteins potentially implicated in our subject's sudden unexplained death. ATP, adenosine triphosphate; MYBPC, myosin binding protein C; RyR, ryanodine receptor; TTN, titin; VLCAD, very long‐chain acyl‐CoA dehydrogenase; VLCFA, very long‐chain fatty acids

According to WebGestalt algorithms, when testing the phenotype association enrichment analysis for all the 11 genes mutated, *MYBPC3*, *TTN*, *ACADVL,* and *RYR2* resulted significantly associated with the cardiac arrest phenotype (*p* value = 2.81e‐06; FDR = 1.47e‐02) (Figure 1a).

## 5 DISCUSSION

Epidemiological studies indicate that most SCD in young adults are related to inherited cardiac diseases (Bagnall et al., 2016; Hayashi et al., 2015). Cardiomyopathies like hypertrophic (HCM), dilated (DCM), and arrhythmogenic (AC) cardiomyopathies are due to mutations in genes coding for structural proteins, and are associated with morphologic abnormalities of the heart. On the contrary, channelopathies, such as long QT syndrome (LQTS), short QT syndrome (SQTS), Brugada syndrome (BS), and catecholaminergic polymorphic ventricular tachycardia (CPVT), are caused by mutations in genes encoding membrane ion channels or cellular structures that affect Ca^2+^ availability, and are associated with life‐threatening arrhythmias, even in the absence of a structural heart disease (Saenen et al., 2015).

We report a case in which macroscopic and microscopic forensic examination were negative. To date, more than 100 inherited genes have been associated with susceptibility to SCD, and the lack of a clear diagnosis after autopsy in SUD makes it difficult to identify candidate genes to test for pathogenic mutations. Differently from Sanger sequencing, NGS technologies like WES represent time‐ and cost‐efficient tools to perform a wide screening of potential pathogenic mutations in the coding region of the genome, making possible a true “molecular autopsy” (Lahrouchi, Behr, & Bezzina, 2016). By performing WES, we were able to reevaluate an unsolved case of SUD of 14 years before, where we detected 11 potentially pathogenic variants, four likely dangerous, based on our analysis. Since the number of genetic variants found in a single individual can be huge, NGS results deserve careful interpretation. To evaluate the pathogenicity of a genetic variant most studies have relied on data derived from online databases and in silico analyses (Hata et al., 2016; Hata, Kinoshita, & Nishida, 2017; Wang et al., 2015). Given the discordance often provided, ≥2 in silico analyses are usually recommended. Nonetheless, the identification of a single potentially dangerous variant does not necessarily confirm a disease; indeed, healthy individuals can carry genetic variants related to syndromes/diseases associated with SCD (Le Scouarnec et al., 2015). Therefore, when applying NGS techniques to cases of SUD, it is fundamental to put the detected variants in the appropriate pathophysiological context by examining their potential involvement in plausible biological pathways.

MYBPC3:p.R419C (rs368770848) is a non‐conservative amino acid substitution likely impairing structure and/or function of myosin binding protein C (MYBPC), a protein modulating muscle contraction. This variant was found in two subjects with HCM (data from ClinVar Database), but its clinical significance is still uncertain. Nonetheless, carriers of *MYBPC3* mutations seem to hold a higher risk of SCD (Calore et al., 2015). TTN:p.R3941C missense variant (rs397517804) was identified in an adult with ventricular tachycardia and possible diagnosis of DCM/AC, and in a teenager with SCD and features of HCM (data from ClinVar Database). However, in our subject no structural findings directly linked to hypertrophy were found. Our subject also presented the variant ACADVL:p.V383A, which is classified as pathogenic for very long‐chain acyl‐CoA dehydrogenase (VLCAD) deficiency in an autosomal recessive manner. Functional analysis found that ACADVL:p.V383A was associated with approximately 20% residual enzyme activity compared to wild type (Goetzman et al., 2007). VLCAD deficiency has been linked to sudden death in infants. However, there are no reports of arrhythmias or sudden death in adults heterozygous for the ACADVL:p.V383A variant. The variant RYR2:p.K4056N has not been previously reported in the available databases. Mutations in this gene are strongly associated with CPVT (Tang, Tian, Wang, Fill, & Chen, 2012) and can lead to lethal arrhythmias due to aberrant activation of the ryanodine receptor 2 (RyR2), which is responsible for Ca^2+^ release from the sarcoplasmic reticulum into the cytoplasm during cardiac excitation–contraction coupling. RYR2:p.K4056N is absent in control population databases (MAF = 0) and is located in domain III (channel region, well conserved across species), a “hot spot” for CPVT mutations. Thus, the mutation could alter the functional characteristics of the RyR2 channel (Figure 1b).

The WebGestalt analytical approach allows to assess the probability that a combination of multiple mutated genes may affect a specific pathophysiological process or determine a particular phenotype. Using WebGestalt algorithms, in the present case, the combination of *MYBPC3*, *TTN*, *ACADVL,* and *RYR2* increased significantly the likelihood of cardiac arrest. Based on these results, we speculate that a cardiac arrhythmia due to the identified RyR2 sequence alteration (p.K4056N) might have contributed to the death in our SUD victim. Moreover, the combination of the other three genetic variants (TTN:c.11821C>T; MYBPC3:c.1255C>T; ACADVL:c.848T>C) may have increased the pro‐arrhythmic susceptibility, eventually favoring the events leading to our subject's SCD. This is in accordance with recent studies suggesting SCD as a complex disease where multiple genetic variants may cumulatively contribute to its manifestations (Wang et al., 2015). The absence of a clear phenotype at autopsy examination does not exclude the potential contribution of TTN, MYBPC3, and ACADVL to the electrical instability that caused our subject's SCD. Actually, there are reports of SCD cases with cardiomyopathy‐related mutations in absence of a distinct phenotype at pathological examination (Lahrouchi et al., 2017). Animal studies support the notion that in these cases the mutated protein may determine subtle or localized structural abnormalities not detectable by autopsy or, alternatively, affect the electrical homeostasis before the onset of the cardiomyopathy phenotype (Rizzo et al., 2012).

“Molecular autopsy” through NGS techniques in cases of SUD may allow the identification of rarer pathogenic mutations, which are not routinely evaluated by traditional Sanger sequencing and can be the subject of further screening in the victims’ relatives. Moreover, WES and bioinformatic analysis provide the possibility to unveil novel plausible genetic substrates underlying the observed phenotypes, and can guide the design of the functional studies needed to achieve a conclusive proof of pathogenicity (Rodenburg, 2018). A formal proof of causality between genetic variants and phenotype usually requires either pedigree assessment or functional validation. Pedigree assessment is particularly valuable because it can also dramatically impact on clinical management of at‐risk family members. However, when reevaluating old cases of SUD, as for our subject, it is not always possible to track down close relatives on whom to perform genetic testing. Functional validation studies of genetic variants are a powerful, though often time‐consuming and expensive tool to obtain evidence for pathogenicity. They consist of either molecular (mRNA expression and localization, protein localization, protein‐protein, and protein‐DNA interaction) or biological assays (cell lines and animal models) allowing the evaluation of gene functions at a molecular level or demonstrate the relationship between a genetic entity and the observed phenotype. In this regard, induced pluripotent stem cells (iPSCs), that is, reprogrammed somatic cells into pluripotent stem cells, have emerged as an effective approach for the investigation of the molecular pathophysiology and new therapeutic targets in cardiomyopathies and channelopathies (Bruyneel, McKeithan, Feyen, & Mercola, 2018). Though iPSCs have been so far largely utilized for investigations in monogenic diseases, their application may be promising also for validation of combination of gene variants, which could underlie complex cases of SUD. Indeed, it would be conceptually possible to use CRISPR/Cas9 genome editing approach in iPSCs to remove a single mutation from the supposed pathogenic combination of variants, in order to assess the relative contribution of the remaining mutations to the observed phenotype.

As a possible limitation, the quality of DNA extracted from FFPE tissues is lower compared to other biological samples, due to excessively fragmented DNA that preclude its use for traditional Sanger sequencing which relies on reads of longer fragments. However, these samples are the only available ones in retrospective studies, as it is the case. NGS techniques can overcome this limit since the read length is much shorter (on the order of 50–250 base pairs), making FFPE a viable source for nucleic acid interrogation (Bagnall et al., 2016). Nevertheless, to avoid possible artefacts, the variants were confirmed by standard Sanger sequencing.

In conclusion, WES and bioinformatic analysis allow the screening of the whole coding region of the genome in search for causative genetic mutations for sudden death and can be effectively used for the reassessment of unsolved cases of SUD. This molecular autopsy approach can either identify rarer pathogenic variants which can be missed by traditional sequencing techniques or provide novel candidate genetic variants to be tested in functional studies in order to shed new light into the pathophysiology of sudden death.

## CONFLICT OF INTEREST

The authors declare that there are no conflicts of interest.
