# Supplementary material for: Unveiling a sudden unexplained death case by whole exome sequencing and bioinformatic analysis
Source: Mol Genet Genomic Med. 2020 Feb 26;8(4):e1182. doi: 10.1002/mgg3.1182 (PMC7196487; doi:10.1002/mgg3.1182)
Supplement: Supplementary file 1 [file MGG3-8-e1182-s001.docx]

**Supporting Information Table 1.** List of variants after first filtering.

After first filtering we identified 13 variants; including nonsynonymous, frameshift and missense variants and excluding synonymous variants, in a list of 234 cardiovascular related genes, with minor allele frequency (MAF) <0.1. Human reference sequence (GRch37).

| **Chromosome** | **Chromosome**  **start** | **Chromosome end** | **Variant type** | **Reference sequence** | **Variant sequence** | **rsID** | **Gene** | **Gene component** | **Protein impact** | **AA change** | **Global allele frequency** | **Mammalian conservation** |
| --- | --- | --- | --- | --- | --- | --- | --- | --- | --- | --- | --- | --- |
| 1 | 74808631 | 74808631 | SNP (x1) | C | T | rs34521608 | *FPGT-TNNI3K* | CDS | MISSENSE | P-377-L | 0.25 | Yes (5.7) |
| 1 | 237947180 | 237947180 | SNP (x1) | G | T |  | *RYR2* | CDS | MISSENSE | K-4056-N | 0 | No |
| 2 | 21247983 | 21247983 | SNP (x1) | C | T | rs148502464 | *APOB* | CDS | MISSENSE | G-753-E | 0.02 | Yes (5.7) |
| 2 | 167262274 | 167262274 | SNP (x1) | C | T | rs188781935 | *SCN7A* | CDS | MISSENSE | R-1622-Q | 0.15 | Yes (3.6) |
| 2 | 179441295 | 179441295 | SNP (x1) | T | C | rs72646885 | *TTN-AS1; TTN* | INTRON; CDS | MISSENSE | S-20658-G | 0.75 | Yes (4.5) |
| 2 | 179482089 | 179482089 | SNP (x1) | C | T | rs72677237 | *TTN-AS1; TTN* | INTRON; CDS | MISSENSE | R-13340-H | 0.64 | Yes (5.9) |
| 2 | 179615306 | 179615306 | SNP (x1) | G | A | rs397517804 | *TTN-AS1; TTN* | INTRON; CDS | MISSENSE | R-3941-C | 0.01 | Yes (5.5) |
| 2 | 179649066 | 179649066 | SNP (x1) | C | T | rs751157908 | *TTN* | CDS | MISSENSE | G-836-S | 0.01 | Yes (5.5) |
| 3 | 39225585 | 39225585 | SNP (x1) | C | T | rs146284335 | *XIRP1* | CDS | MISSENSE | M-1784-I | 0.71 | No |
| 4 | 114279453 | 114279453 | SNP (x1) | A | C | rs140604600 | *ANK2* | INTRON; CDS | MISSENSE | T-3227-P | 0.01 | No |
| 6 | 76576730 | 76576730 | SNP (x1) | C | T | rs755596824 | *MYO6* | CDS | MISSENSE | R-618-W | 0.01 | Yes (4.5) |
| 7 | 128489424 | 128489424 | SNP (x1) | C | T | rs780829334 | *FLNC* | CDS | MISSENSE | T-1664-M | 0.01 | Yes (5.6) |
| 11 | 47364668 | 47364668 | SNP (x1) | G | A | rs368770848 | *MYBPC3* | CDS | MISSENSE | R-418-C | 0.01 | Yes (4.7) |
| 14 | 23844979 | 23844979 | SNP (x1) | C | T | rs1124053 | *IL25* | CDS | MISSENSE | R-126-W | 0.59 | Yes (3.6) |
| 15 | 57896482 | 57896482 | SNP (x1) | C | A | rs142253131 | *GCOM1* | CDS | MISSENSE | L-31-I | 0.05 | Yes (3.3) |
| 16 | 7743337 | 7743337 | SNP (x1) | C | T |  | *RBFOX1* | CDS | MISSENSE | A-382-V | 0 | Yes (5.9) |
| 17 | 7125591 | 7125591 | SNP (x1) | T | C | rs113994167 | *ACADVL* | CDS | MISSENSE | V-283-A | 0.14 | Yes (5.2) |
| 22 | 40075733 | 40075733 | SNP (x1) | C | A | rs56656729 | *CACNA1I* | CDS | MISSENSE | L-1766-M | 0.98 | No |

AA, amino acid; CDS, coding DNA sequence; SNP, single-nucleotide polymorphism.
