# Supplementary material for: Unveiling a sudden unexplained death case by whole exome sequencing and bioinformatic analysis
Source: Mol Genet Genomic Med. 2020 Feb 26;8(4):e1182. doi: 10.1002/mgg3.1182 (PMC7196487; doi:10.1002/mgg3.1182)
Supplement: Supplementary file 2 [file MGG3-8-e1182-s002.docx]

**Supporting Information Table 2:** List of identified variants with probable pathogenic effects.

| Variant type | Genomic  change | rsID | Gene | Protein impact | AA change | ACMG Classification | Identified criteria | ClinVar | gnomAD frequency | DANN | Mutation Taster | FATHMM | Mutation Assessor | PolyPhen-2 | SIFT | PROVEAN | GERP++ |
| --- | --- | --- | --- | --- | --- | --- | --- | --- | --- | --- | --- | --- | --- | --- | --- | --- | --- |
| SNP  (x1) | 1-237947180G>T |  | *RYR2* | MISSENSE | K4056N | VUS | PM2  PP2  PP3 | . | 0 | 0.998 | D | D | M | D | D | D | 0,61 |
| SNP  (x1) | 2-21247983C>T | rs148502464 | *APOB* | MISSENSE | G753E | VUS | PM2 | . | 4.07e-06 | 0.993 | D | T | M | D | T | N | 5.7 |
| SNP  (x1) | 2-167262274C>T | rs188781935 | *SCN7A* | MISSENSE | R1622Q | VUS | PP3 | . | 0.00156 | 0.999 | D | D | M | P | D | D | 3.6 |
| SNP  (x1) | 2-179615306G>A | rs397517804 | *TTN* | MISSENSE | R3941C | VUS | BP1 | Uncertain significance | 1.23e-05 | 0.998 | D | T | . | D | D | N | 5.5 |
| SNP  (x1) | 6-76576730C>T | rs755596824 | *MYO6* | MISSENSE | R618W | VUS | PM2  PP3 | . | 1.63e-05 | 0.998 | D | T | M | D | D | D | 4.5 |
| SNP  (x1) | 7-128489424C>T | rs780829334 | *FLNC* | MISSENSE | T1664M | VUS | PP3 | Uncertain significance | 6.91e-05 | 0.999 | D | D | L | D | D | D | 5.6 |
| SNP  (x1) | 11-47364668G>A | rs368770848 | *MYBPC3* | MISSENSE | R418C | VUS | PM1  PM2  PP3 | Uncertain significance | 2.49e-05 | 0.999 | D | T | M | . | D | D | 4.7 |
| SNP  (x1) | 17-7125591T>C | rs113994167 | *ACADVL* | MISSENSE | V306A | VUS | PM1  PP3  PP5 | Pathogenic | 0.00128 | 0.997 | A | D | L | . | D | D | 5.2 |
| SNP  (x1) | 14-23844979C>T | rs1124053 | *IL25* | MISSENSE | R126W | VUS | PP3 | . | 0,0064 | 0,999 | D | T | M | D | D | D | 3.6 |
| SNP  (x1) | 15-57896482C>A | rs1124053 | *GCOM1* | MISSENSE | L31I | VUS | PP3 | . | 0,0005 | 0.993 | D | T | M | D | D | N | 3.4 |
| SNP  (x1) | 16-7743337C>T |  | *RBFOX1* | MISSENSE | A382V | VUS | PP3 PM2 | . | 0 | 0,998 | D | T | . | D | D | N | 5.9 |

MutationTaster: A=disease_causing_automatic; D=disease_causing; N=polymorphism; P=polymorphism_automatic.

FATHMM: D=deleterious; T=tolerated.

MutationAssessor: H=high; M=medium; L=low; N=neutral.

PolyPhen-2: D=probably damaging (≥0.957); P=possibly damaging (0.453≤pp2_hdiv≤0.956); B=benign (pp2_hdiv≤0.452).

SIFT: D=deleterious (sift≤0.05); T=tolerated (sift>0.05).

PROVEAN: D=deleterious; N=neutral.

AA, amino acid; ACMG, American College of Medical Genetics and Genomics; DANN, Deleterious Annotation of genetic variants using Neural Networks; FATHMM, Functional Analysis Through Hidden Markov Models; GERP, Genomic Evolutionary Rate Profiling; PolyPhen-2, Polymorphism Phenotyping v2; PROVEAN, Protein Variation Effect Analyzer; SIFT, Sorting Intolerant From Tolerant; SNP, single-nucleotide polymorphism; VUS, variant of uncertain significance.
